# A novel downlink scheduling strategy for traffic communication system based on TD-LTE technology

**DOI:** 10.1186/s40064-016-3323-x

**Published:** 2016-09-21

**Authors:** Ting Chen, Xiangmo Zhao, Tao Gao, Licheng Zhang

**Affiliations:** 1 School of Information Engineering, Chang’an University, Xi’an, 710064 China; 2 School of Electrical and Information Engineering, University of Sydney, Sydney, NSW 2006 Australia

**Keywords:** Traffic communication system, TD-LTE, Scheduling, AMC, QoS, OFDMA

## Abstract

There are many existing classical scheduling algorithms which can obtain better system throughput and user equality, however, they are not designed for traffic transportation environment, which cannot consider whether the transmission performance of various information flows could meet comprehensive requirements of traffic safety and delay tolerance. This paper proposes a novel downlink scheduling strategy for traffic communication system based on TD-LTE technology, which can perform two classification mappings for various information flows in the eNodeB: firstly, associate every information flow packet with traffic safety importance weight according to its relevance to the traffic safety; secondly, associate every traffic information flow with service type importance weight according to its quality of service (QoS) requirements. Once the connection is established, at every scheduling moment, scheduler would decide the scheduling order of all buffers’ head of line packets periodically according to the instant value of scheduling importance weight function, which calculated by the proposed algorithm. From different scenario simulations, it can be verified that the proposed algorithm can provide superior differentiated transmission service and reliable QoS guarantee to information flows with different traffic safety levels and service types, which is more suitable for traffic transportation environment compared with the existing popularity PF algorithm. With the limited wireless resource, information flow closed related to traffic safety will always obtain priority scheduling right timely, which can help the passengers’ journey more safe. Moreover, the proposed algorithm cannot only obtain good flow throughput and user fairness which are almost equal to those of the PF algorithm without significant differences, but also provide better realtime transmission guarantee to realtime information flow.

## Background

As an important part of Intelligent Transportation System (ITS), traffic communication system (Papadimitratos et al. 2009) could ensure all the traffic participants communicating with each other smoothly; provide security early warning and effective navigation for the monitored vehicles, and other multimedia or mobile Internet application services for passengers. Therefore, further strengthening construction of traffic communication system to make passengers’ journey safer and more comfortable, is one of basic guarantee measures for intelligent transportation industry’s sustainable development.

There are mainly two realistic modes to release various traffic information in traffic communication system: the first mode is where all the traffic participants organize themselves and deliver traffic information either single-hop or multi-hop; the second mode is where all the traffic participants deliver traffic information to each other via a roadside base station which has total control of the whole system. Jurgen () give a summary of some characteristics of two modes. The majority of researches on traffic communication system focus on the first mode based on ad hoc networks or sensor networks (Sichitiu and Kihl 2008; Liang et al. 2015; Paier et al. 2010; Li 2010; Biswas et al. 2006). Since the first mode has a limit application scope, such as collision avoiding, driving assistant or vehicle’s vertical control, this paper pays more attention to the latter mode. As the representative of the 4th generation mobile communication technologies, TD-LTE (Astely et al. 2010; Araniti et al. 2013) has better spectrum efficiency and higher transmission rate. For example, with system bandwidth of 20 MHz and eNodeB coverage radius up to 100 km, system peak rate of TD-LTE can be up to 100 Mbps for the downlink, and 200 Mbps for the uplink, respectively; Since TD-LTE has a flat system architecture, the one-way transmission delay in its user plane is less than 5 ms, and the system transition delay in its control plane from sleep state to activated state is less than 50 ms, and from resident state to activated state is less than 100 ms, respectively. TD-LTE system adopts an advanced communication technology called orthogonal frequency division multiple access (OFDMA), which can divide wireless resources into some independent resources blocks (RBs) in both the time as well as frequency domain, and let scheduler adaptively decide which RB would be allocated to a specific user at a given time according to the instantaneous value of schedule function for mitigating the traditional fading effects.

In our paper, we design a novel TD-LTE downlink scheduling strategy used in the proposed traffic communication system, according to the characteristics of various traffic information flows. This proposed scheduling strategy can differentiate traffic information flows with different safety warning levels and different service types, and then give each user the priority right to schedule the traffic information flow with the highest safety warning level, and at meantime, meet different service type’s QoS requirements as much as possible, such as the maximum waiting delay, the target packet error rate, etc. We evaluate the performance of the proposed strategy when used for traffic communication system. Simulation scenarios are developed in LTE-Sim (Piro et al. 2011; http://telematics.poliba.it/index.php/en/lte-sim), one of the few open source simulators available for performance simulations of LTE systems. The paper is organized as follows. In “Literature review” section, the corresponding literatures are presented. In “System composition and scheduling strategy” section, we propose the composition and scheduling model of traffic communication system based on TD-LTE and overview the characteristics of various traffic information flows. In “Scheduling modeling and algorithm” section, we propose a novel downlink scheduling strategy and its corresponding algorithm suitable for the TD-LTE traffic communication system according to the characteristics of various traffic information flows. In “Simulating and performance evaluation” section, we introduce the simulation settings and present the evaluation results. Finally, our conclusions are summarized in “Conclusion” section.

## Literature review

Recently, some researchers begin to pay close attention to the application of TD-LTE, since it will provide more robust communication link support if introduced into traffic communication system (Abid et al. 2012; Vinel 2012). The concept car based on 4G technologies is presented for various applications like infotainment, diagnostics and navigation, and it is shown that the system works very well up to speeds of 140 km/h when working at the transmission rate of 10 Mbps (Mosyagin 2010). The problem of power and sub-carrier allocation in OFDMA systems has been the subject of many researches (Zhu 2012; Del-Castillo et al. 2013; Srikanth et al. 2012). Capozzi et al. (2013) summarize the key design issues of the downlink scheduling in the TD-LTE cellular networks and give a survey on the current research status with a performance comparison of the most well-known techniques of LTE scheduling, but without considering any scheduling application in the traffic communication system. Kihl et al. (2012) study the downlink scheduling strategies for traffic safety applications, but only evaluating the performance of the existing scheduling strategies.

Although there have been many scheduling algorithms of wireless communication, current TD-LTE commercial system still used the most classical three scheduling algorithms for commercial business, respectively is Round Robin (RR), Maximum Throughput (MT), and Proportional Fair (PF; Capozzi et al. 2013; Kihl et al. 2012; Chen 2011). RR algorithm can make all users get RBs in fixed order with equal time interval, which can ensure them access to TD-LTE system with short-term as well as long-term fairness, but the system throughput is relatively low. At every scheduling moment, MT algorithm can assign RBs to users which could achieve its own maximum throughput, and then maximize the system throughput, but users far from eNodeB are difficult to get any RB because of its bad channel condition, which would cause “starvation phenomenon”. PF algorithm is a compromise of RR and MT, which can make users obtain RBs with long-term fairness, and achieve system throughput as high as possible at meantime, therefore, PF can be used as the preferred scheduling algorithm for practical TD-LTE traffic communication system. However, when allocating wireless resource to the related VS terminals within the coverage of the eNodeB, these above three classical scheduling algorithms only considered system throughput and user equality, did not consider whether the traffic information flow’s transmission performance could meet the requirements of safety warning priority and delay tolerance. Since it is very important for VS terminals to reduce life or property loss, and improve traffic efficiency, through obtaining all kinds of traffic information timely and reliably, a new algorithm should be proposed to improve the existing PF algorithm for providing various traffic information flows with differentiated quality of service (QoS) guarantee.

## System composition and scheduling strategy

**Fig. 1:**
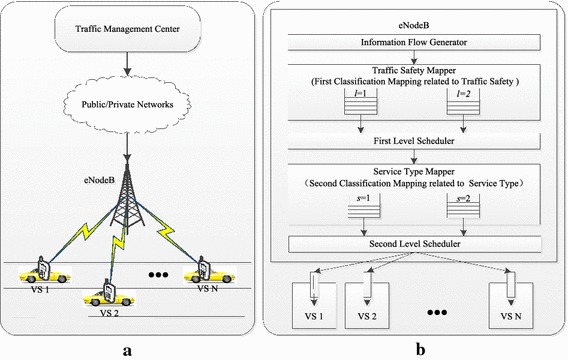
TD-LTE traffic communication system and its scheduling model. **a** System composition, **b** Scheduling strategy

In the proposed TD-LTE traffic communication system (Fig. 1a), various kinds of information flows are firstly transmitted by traffic management center to TD-LTE base station called eNodeB, through the public or private network, and then, again by eNodeB to corresponding vehicle subscriber (VS) terminals quickly and reliably. In this case, VSs within coverage of eNodeB need not to be aware of each other, since eNodeB will have total control of the proposed system and allocate TD-LTE wireless resources to specific users at the given time for transmitting different information flows with certain importance weights related to traffic safety and service type.

When eNodeB transmits information flows to a VS terminal, one data bearer connection is established firstly between the equivalent media access control layers of eNodeB and this VS terminal. Once the connection is established, scheduling process will be started to provide QoS guarantee for information flows’ transmission. As shown in Fig. 1b, the proposed scheduling strategy is designed to schedule information flow packets at every scheduling time according to their traffic safety characteristics as well as their service type characteristics:

1. *Traffic safety mapping and the first scheduling* There are two kinds of information flow according to the quality of their relationships with traffic safety: one can provide safe high-effect transportation guarantee to the monitored VSs, such as security early warning and effective navigation, which should be issued to the related VS terminals quickly and reliably, so as to avoid and reduce the number of traffic accidents effectively through an active way of warning, or improve the traffic efficiency through active guidance or independent inquiry for auxiliary driving; the other one can provide various multimedia or mobile Internet application services on demand, such as VoIP, video conference, web browsing, etc., which should be initiated by the related VS terminals, so as to enrich passengers’ driving experience. Obviously, it is more urgent to transmit the first kind of information flows as soon as possible which closely related to traffic safety if compared with the other kind of information flows. In order to provide differentiated transmission services to the above mentioned two kinds of information flows, packets generated from the information flow generator will be firstly classified into two classes based on traffic safety characteristics by traffic safety mapper: information flow packets closely related to traffic safety will be mapped into the first buffer queue in a sequence and associated with a larger value of traffic safety importance weight; information flow packets aiming at enriching passengers’ driving experience will be mapped into the second buffer queue in a sequence and associated with a smaller value of traffic safety importance weight. At each scheduling time, the first level scheduler will decide the scheduling order of traffic safety buffer queues’ head of line (HOL) packets: HOL packet from the first buffer queue with larger weight will be always delivered to the next service type mapper prior to HOL packet from the second buffer queue with smaller weight.
2. *Service type mapping and the second scheduling* Small amount of data burst flows were usually adopted for transmitting some traffic safety information in the past transportation system, but nowadays are unable to meet the needs of ITS information growth. For the future ITS, massive information flows in different forms of service type, such as audio, video, as well as data burst will be exchanged not only for enriching passengers’ driving experience, but also for providing safe high-effect transportation guarantee in modern traffic communication system. Therefore, in the proposed system, after the first scheduling, packets with different traffic safety importance weights will be further classified based on their service type characteristics and delivered into corresponding buffer queues in a sequence by service type mapper. There are two kinds of buffer queues defined in our paper, which are real-time service type and non-real-time service type, respectively. Every service type buffer’s HOL packet will be once again associated with corresponding service type importance weight, which adaptively decided by: HOL packet’s service type QoS requirements, such as transmission delay limitation threshold, target packet error rate, etc.; the instant practical QoS performance of information flow which that HOL packet belongs to, such as HOL packet’s instant waiting time in its service type buffer, HOL packet instant data transmission rate, etc. At every scheduling time, the second level scheduler will adaptively decide the scheduling order of every service type buffer queue’s HOL packet: HOL packets with lager importance weights related to traffic safety and service type are always delivered to the target VS terminals prior to that with smaller importance weights.

Overall, the proposed scheduling strategy will perform two classification mappings and two schedulings to provide differentiated scheduling service for various kinds of information flows. With limited wireless resource, this proposed scheduling strategy is aiming at delivering information flows which closely related to traffic safety from eNodeB to its target VS terminal timely, as well as making various information flows meet some QoS requirements of their own service type. It can be seen obviously that importance weights related to traffic safety and service type for each buffer queue’s HOL packet are very critical when making decision of HOL packets’ corresponding scheduling order. The scheduling function modeling of those importance weights is shown in the “System composition and scheduling strategy” section.

## Scheduling modeling and algorithm

**Fig. 2:**
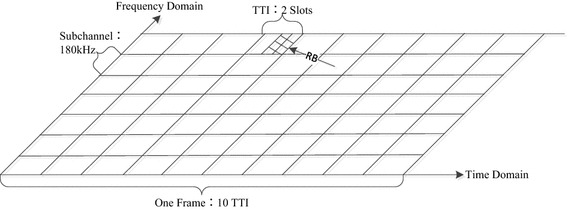
Wireless resource allocation of TD-LTE traffic communication system

For utilizing multi-user diversity and increasing scheduling flexibility, OFDMA, in which multiple VSs can simultaneously share all limited sub-carriers, is employed in the proposed traffic communication system. OFDMA can provide every VS terminal with more flexible wireless resource access in both time and frequency domain. In time domain, wireless resources of TD-LTE system are divided into a series of transmission time intervals (TTIs), each TD-LTE frame contains 10 successive TTIs, each TTI is last 1 ms, including two slots; in frequency domain, the whole system bandwidth is divided into a series of sub-channels, each sub-channel is 180 kHz, including 12 consecutive sub-carriers. As illustrated in Fig. 2, RB is the smallest time–frequency resource allocation unit, which consists of two slots in time domain, and one sub-channel in frequency domain. Each RB can be allocated to only one VS terminal at every scheduling time to bear one kind of information flow from eNodeB. Since the sub-channel bandwidth is fixed, different system bandwidths are with different numbers of RBs, for example, 5 MHz and 10 MHz system bandwidth are with 25 RBs and 50 RBs, respectively.

Suppose there are N VSs in the proposed traffic communication system, and at each scheduling time, there are K RBs that can bear information flow transmission. At the given scheduling time *t*, scheduler should seek the best matching identifier $C_{l, \, s, \, n, \, k} \left( t \right)$ among RBs, various information flows and VSs, which can maximize system throughput of the proposed traffic system is as flows:

1 $$C_{l, \, s, \, n, \, k} \left( t \right) = \arg \left\{ {\mathop {\hbox{max} }\limits_{l, \, s, \, n, \, k} \sum\limits_{l = 1}^{2} {\sum\limits_{s = 1}^{2} {\sum\limits_{k = 1}^{\text{K}} {\sum\limits_{n = 1}^{\text{N}} {C_{l, \, s, \, n, \, k} \left( t \right)R_{l, \, s, \, n, \, k} \left( t \right)} } } } } \right\}$$

and at the same time, it must satisfy the following conditions:

2 $$\tau_{s = 1,n} \left( t \right) \le T_{s = 1}$$

3 $$\xi_{n} = \frac{ 1}{\text{N}}$$

where $C_{l, \, s, \, n, \, k} \left( t \right)$ is defined as the following:

4 $$\left. {\left\{ {\begin{array}{*{20}c} {C_{l, \, s, \, n, \, k} \left( t \right) \in \left\{ {0,1} \right\}} \\ {\sum\limits_{k = 1}^{\text{K}} {C_{l, \, s, \, n, \, k} \left( t \right)} = 1} \\ \end{array} } \right\}} \right|\begin{array}{*{20}c} \quad{\forall l = \left\{ {1,2} \right\}, \, \quad\forall s = \left\{ {1,2} \right\}, \, \quad\forall k = \left\{ {1,2, \ldots ,{\text{K}}} \right\}, \, \quad\forall n \in \left\{ {1,2, \ldots ,{\text{N}}} \right\}} \\ \end{array}$$

$R_{l, \, s, \, n, \, k} \left( t \right)$ is the *k*th RB’s transmission rate if bearing the information flow with the *l*th traffic safety and *s*th service type delivered to the *n*th VS; Eq. (4) can make sure each RB only be allocated to one VS, when and only when the *n*th VS with the *l*th traffic safety and *s*th service type is matched with the *k*th RB, it has $C_{l, \, s, \, n, \, k} \left( t \right) = 1$. $\tau_{s = 1,n} \left( t \right)$ is the instantaneous waiting time of HOL packet with real-time service type, $T_{s = 1}$ is the transmission delay limitation threshold of packet with real-time service type; Eq. (2) can make sure real-time information flows meet their time delay requirement. $\xi_{n} \left( t \right)$ means the proportion of wireless resource allocated to the *n*th VS in the whole available wireless resources, often satisfying Eq. (5):

5 $$\sum\limits_{n = 1}^{N} {\xi_{n} \left( t \right)} = 1$$

generally, for $\forall n_{1} ,n_{2} \in \left\{ {1,2, \ldots ,{\text{N}}} \right\}$, and $n_{1} \ne n_{2}$, it has:

6 $$R_{{n_{1} }} :R_{{n_{2} }} = \xi_{{n_{1} }} :\xi_{{n_{2} }}$$

in Eq. (6), $R_{n}$ is the *n*th VS’s throughput which can be calculated from Eq. (7):

7 $$R_{n} \left( t \right) = \sum\limits_{l = 1}^{2} {\sum\limits_{s = 1}^{2} {\sum\limits_{k = 1}^{K} {C_{l, \, s, \, n, \, k} \left( t \right)R_{l, \, s, \, n, \, k} \left( t \right)} } }$$

Equation (6) means the transmission rate ratio of any two VSs is equal to the wireless resource ration obtained by themselves, which can be predefined according to various system requirements for different profits. If making each VS obtain system wireless resource fairly, it has $R_{{n_{1} }} :R_{{n_{2} }} = 1 :1$ from which the Eq. (3) can be deduced.

From the above, the ideal scheduling principle is maximizing the system throughput, meeting the time-delay requirement as well as obtaining system VSs’ absolute fairness. However, it is a NP-hard combination optimizing problem with nonlinear limitation conditions which can be hard to get the optimal solution, which forces us to simplify the scheduling algorithm and make it easy to implement in the practical condition.

As illustrated in Fig. 1, if a packet queued in the first buffer queue or second one of traffic safety mapper, its traffic safety importance level are respectively defined as *l* = 1, or *l* = 2 and then the packet will be delivered by the first level scheduler to the next mapper according to its corresponding safety importance level. Furthermore, if this packet further delivered into the first buffer queue or second one in service type mapper, its service type are respectively defined as *s* = 1, or *s* = 2. At the given scheduling time *t*, if the scheduling importance weight of HOL packet with the $l^{{\prime }}$th traffic safety level and $s^{{\prime }}$th service type which delivered from eNodeB to the $n^{{\prime }}$th VS via the $k^{{\prime }}$th RB, are of the largest value, the second scheduler will deliver the information flow which that HOL packet belongs to as depicted in Eq. (8):

8 $${\text{flow}} = \mathop { \arg }\limits_{{l^{{\prime }} ,s^{{\prime }} ,n^{{\prime }} ,k^{{\prime }} }} \left\{ {\begin{array}{*{20}c} {\hbox{max} \omega_{l, \, s, \, n, \, k} \left( t \right)} \\ \end{array} } \right\}$$

which can further deduce Eq. (9):

9 $$C_{{l^{\prime}, \, s^{\prime}, \, n^{\prime}, \, k^{\prime}}} \left( t \right) = 1$$

Therefore, the second level scheduler will periodically match each RB with proper information flow which delivered to different VSs according to the function of the instant scheduling importance weight.

Since the typical PF algorithm can find a good trade-off between requirements on fairness and spectral efficiency, the PF algorithm factor is introduced into the scheduling importance weight. At a given scheduling time *t*, if eNodeB uses the *k*th RB (*k* = 1, 2, … K) to transmit the HOL packet with the *l*th traffic safety importance level and *s*th service type to the *n*th VS terminal (*n* = 1, 2, … N), its instant scheduling importance weight can be proposed to model as Eq. (10):

10 $$\omega_{l, \, s, \, n, \, k} \left( t \right) = \omega_{l} *\omega_{s,n,k} \left( t \right) * \psi_{n, \, k}^{\text{PF}} \left( t \right)$$

In Eq. (10), $\psi_{n,k}^{\text{PF}} \left( t \right) \in \left( {0,1} \right]$ is the PF algorithm factor if the *k*th RB is selected to transmit information flow to the *n*th VS, which can be expressed as Eq. (11):

11 $$\psi_{n,k}^{\text{PF}} \left( t \right) = \left\{ {\begin{array}{*{20}l} {\frac{{R_{n,k} \left( t \right)}}{{\bar{R}_{n} \left( {t - 1} \right)}}} & {R_{n,k} \left( t \right) < \bar{R}_{n} \left( {t - 1} \right)} \\ 1 & {R_{n,k} \left( t \right) \ge \bar{R}_{n} \left( {t - 1} \right)} \\ \end{array} } \right.$$

where $R_{n,k} \left( t \right)$ is the expected transmission data rate of information flow delivered to the *n*th VS terminal via the *k*th RB at the scheduling time *t*, $\bar{R}_{n} \left( {t - 1} \right)$ is the past average throughput obtained by the *n*th VS terminal until previous scheduling time $\left( {t - 1} \right)$. The bigger the value of $R_{n,k} \left( t \right)$ is, or the smaller the value of $\bar{R}_{n} \left( {t - 1} \right)$ is, the bigger the value of PF algorithm factor is, furthermore the higher the possibility of allocating the *k*th RB to the *n*th VS terminal is. $\bar{R}_{n} \left( t \right)$ is expressed as Eq. (12):

12 $$\bar{R}_{n} \left( t \right) = \alpha \bar{R}_{n} \left( {t - 1} \right) + \left( {1 - \alpha } \right)R_{n} \left( t \right),\quad \omega \in \left( {0,1} \right]$$

where $R_{n} \left( t \right)$ is the transmission data rate obtained by the *n*th user at time *t*, and the regulatory parameter $\alpha$ in Eq. (12) can be calculated in Eq. (13):

13 $$\alpha = 1 - \frac{1}{{T_{\text{W}} }}$$

where $T_{\text{W}}$ is the time window over which fairness wants to be imposed, according to the relation (Chen 2011).

In Eq. (10), $\omega_{l} \in \left( {0,1} \right]$ is a constant which represents HOL packet’s traffic safety importance weight and $\omega_{l = 1} > \omega_{l = 2}$ can make eNodeB provide the priority scheduling chance to those information flows which close related to traffic safety ($l = 1$).

In Eq. (10), $\omega_{s, \, n, \, k} \left( t \right) \in \left( {0,1} \right]$ is a variable which represents HOL packet’s service type importance weight defined in Eq. (14):

14 $$\omega_{s, \, n, \, k} \left( t \right) = \varphi_{s,n} \left( t \right) * \frac{{R_{s,n,k} \left( t \right)}}{{R_{k}^{{\max} } \left( t \right)}}$$

where $\varphi_{s,n} \left( t \right)$ is the delay guarantee factor, which are of different values for different service types as presented in Eqs. (15) and (16):

15 $$\begin{array}{*{20}c} {\alpha_{s = 1,n} \left( t \right) = \left\{ {\begin{array}{*{20}l} {\frac{{\Delta T_{s = 1} }}{{T_{s = 1} - \tau_{s = 1,n} \left( t \right)}}} & {\Delta T_{s = 1} \le T_{s = 1} - \tau_{s = 1,n} \left( t \right)} \\ 1 & {\Delta T_{s = 1} > T_{s = 1} - \tau_{s = 1,n} \left( t \right)} \\ \end{array} } \right.} &\quad {{\text{if}}\;s{ = 1}} \\ \end{array}$$

16 $${\alpha_{s = 2,n} \left( t \right) = 1 \quad {\text{if}}\;s= {2}}$$

For real-time information flow, $\tau_{s = 1,n} \left( t \right)$ is the instantaneous waiting time of HOL packet, $T_{s = 1}$ is the transmission delay limitation threshold of HOL packet, $\Delta T_{s = 1} \in \left( {0,T_{s = 1} } \right]$ is the protected time interval predetermined by the proposed system, usually set as TD-LTE frame length. If $\Delta T_{s = 1} \le T_{s = 1} - \tau_{s = 1,n} \left( t \right)$, namely $\tau_{s = 1,n} \left( t \right) \in \left[ {0,T_{s = 1} -\Delta T_{s = 1} } \right)$, the waiting time of HOL packet with the real-time service can satisfy the target transmission delay requirement $T_{s = 1}$ perfectly, so the second level scheduler does not need to give priority scheduling chance to this HOL packet at the scheduling time *t*; on the contrary, If $\Delta T_{s = 1} \ge T_{s = 1} - \tau_{s = 1,n} \left( t \right)$, namely $\tau_{s = 1,n} \left( t \right) \in \left[ {T_{s = 1} -\Delta T_{s = 1} ,T_{s = 1} } \right)$, the waiting time of HOL packet with real-time service in buffer queue will beyond the delay requirements, so the scheduler should schedule this packet as soon as possible at the scheduling time *t*. For non-real-time information flow, there is no transmission delay requirement to HOL packet; therefore the corresponding delay guarantee factor value can be set to 1 which will have no impact on HOL packet’s service type importance weight.

As the service type importance weight, $\omega_{s, \, n, \, k} \left( t \right) \in \left( {0,1} \right]$ is designed not only to obtain lower time delay for real-time service packets, but also obtain other better QoS performances for both non-real-time and real-time service packets such as higher throughput, lower packet error rate, since ${{R_{s,n,k} \left( t \right)} \mathord{\left/ {\vphantom {{R_{s,n,k} \left( t \right)} {R_{k}^{{\max} } \left( t \right)}}} \right. \kern-0pt} {R_{k}^{{\max} } \left( t \right)}}$ are highly related to one of the key TD-LTE technologies, called adaptive modulation coding (AMC). In Eq. (14), $R_{s,n,k} \left( t \right)$ represents the instant transmission rate if the *k*th RB is selected to transmit information flow with the *s*th service type to the *n*th VS terminal, $R_{k}^{{\max} } \left( t \right)$ represents the maximum transmission rate that the *k*th RB can bear. Both $R_{s,n,k} \left( t \right)$ and $R_{k}^{{\max} } \left( t \right)$ are decided by the instant downlink sub-channel quality of the *k*th RB’s from eNodeB to the *n*th VS. If the sub-channel condition (e.g. SNR) is good, the proposed system will use higher modulation mode and coding rate (e.g. 16QAM, 3/4) in order to achieve higher peak rate of $R_{s,n,k} \left( t \right)$ and $R_{k}^{{\max} } \left( t \right)$; If the channel condition is poor, the proposed system will use lower modulation mode and coding rate (e.g. BPSK, 1/2) to ensure the transmission reliability. Therefore, given certain channel quality, AMC technology aims at providing each VS with high transmission rates as much as possible, and at the same time, meeting each information flow’s target packet error rate (PER) at a scheduling time. The implementation details of AMC technology in TD-LTE traffic communication system can be referred to [*], which not be discussed in this paper. ${{R_{s,n,k} \left( t \right)} \mathord{\left/ {\vphantom {{R_{s,n,k} \left( t \right)} {R_{k}^{{\max} } \left( t \right)}}} \right. \kern-0pt} {R_{k}^{{\max} } \left( t \right)}}$ is introduced into HOL packet’s service type importance weight to help system make the best matching between wireless resources and information flows. When there are various information flows destined to different VS terminals, the if using the *k*th RB to bear information flow which delivered to the *n*th VS with the *s*th service type can obtain higher transmission rate with the predefined target PER, the proposed system will preferentially assigned this *k*th RB to that information flow, for the purpose of obtaining much higher system throughput by multi-user diversity.

## Simulating and performance evaluation

**Fig. 3:**
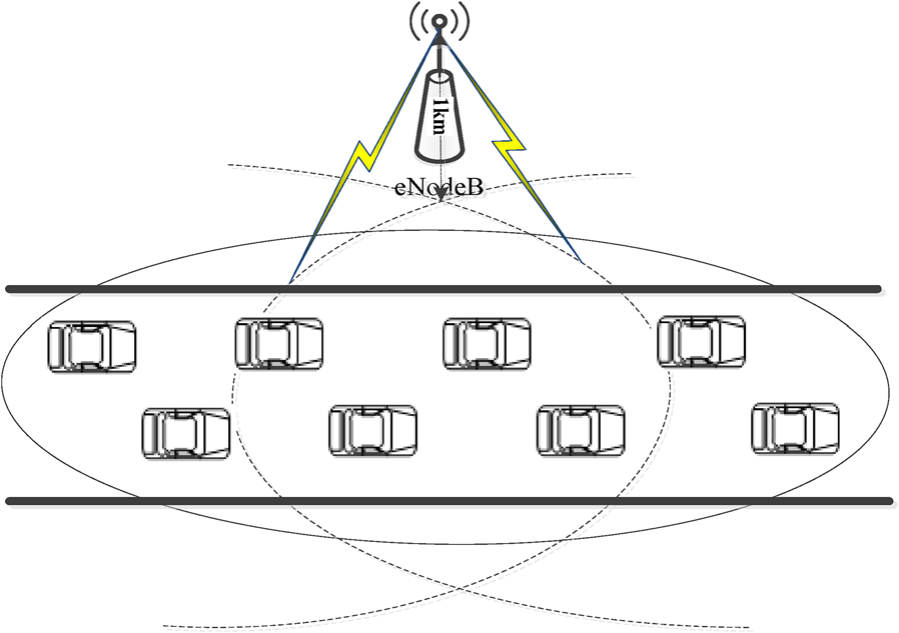
Simulation scenario

The LTE-Sim simulation tool (http://telematics.poliba.it/index.php/en/lte-sim) is used to build time–frequency resource scheduling scenarios of TD-LTE traffic warning system, as illustrated in Fig. 3. The whole communication area with the radius equal to 1 km consists of one eNodeB and several VS terminals. eNodeB is located at the center of this area, and a number of several VS terminals [chosen in the range (5–40)] are uniformly distributed into this area. VSs travel inside this area following the Way Point mobility model in an urban macro cell scenario with average speeds of 30 and 120 km/h, respectively. Other simulation parameters are shown in Table 1. Simulations and performance analysis are performed between the proposed (PRO) algorithm and PF algorithm. Each simulation lasts 100 s. Simulation has been done using a Linux machine with a 2.6 GHz CPU and 4 GB of RAM. There are two realistic scenarios as follows:

**Table 1:** Other simulation parameters

Parameter	Value
Simulation duration	100 s
Physical details	Carrier frequency: 2 GHz
	Bandwidth for the downlink: 10 MHz
	Symbols for TTI: 14
	Sub-frame length: 1 ms
	Sub-carries per RB: 12
	Sub-carrier spacing: 15 kHz
	eNodeB power transmission: 43 dB
	Propagation model: macro-cell urban model
Link adaptation	Modulation schemes: QPSK, 16QAM
	Target BLER: 10^−1^
Control overhead	RTP/UDP/IP with ROCH compression: 3 byte
	MAC and RLC: 5 bytes
	PDCP: 2 bytes
	CRC: 3 bytes
	PHY: 3 symbol
Target delay threshold	100 ms
Channel quality indication	Period: 2 ms
Radio link control	Re-transmission times: 5

1. Scenario I: information flows with different service type:

Each VS terminal receives 3 traffic information flows as video, VoIP, and data burst, which have the same traffic safety importance with either *l* = 1 or *l* = 2. Among them, both video and VoIP are realtime service types with the target block error rate (BLER) threshold $10^{ - 1}$, and data burst is non-realtime service type. We use “highway.yuv” video test sequence, “G.729” audio test sequence and FTP download to simulate Video flow, VoIP flow and data burst, respectively.

**Fig. 4:**
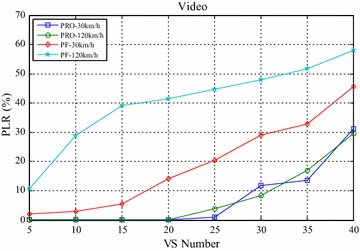
PLR of video

**Fig. 5:**
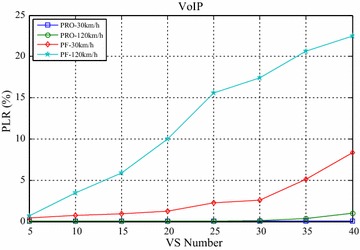
PLR of VoIP

**Fig. 6:**
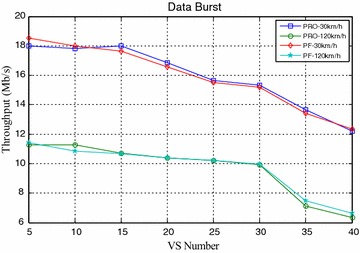
Throughput of data burst

**Fig. 7:**
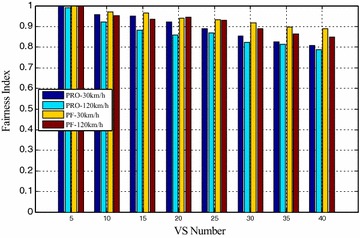
Fairness index of VS terminals

Figures 4 and 5 compared the packet loss rate (PLR) of video flow and VoIP flow respectively. As VS terminals’ number increasing, system traffic load is getting heavier and heavier, and packet loss rate is also increasing. It still worth noting that when VS moving speed increasing, both video and VoIP flow’s PLR are growing, which is caused by sub-frames’ frequent channel condition change when the system making a mistake for selecting wrong modulation level and coding rate. It can be seen clearly that the proposed algorithm achieves better performance for real-time service information flow than PF algorithm. Furthermore, both the video flow and VoIP flow are real-time services, the proposed algorithm can adaptively adjust HOL packets’ scheduling order according to every HOL packet’s waiting time in its own buffer by corresponding service type importance weight $\omega_{s,n,k} \left( t \right)$. The longer the HOL packet’s waiting time of realtime service flow is, the bigger the instant value of $\omega_{s,n,k} \left( t \right)$ is. Scheduler always selects the HOL packet which will be in violation of the delay limitation immediately, give it scheduling priority and deliver it as fast as possible, therefore, the proposed algorithm can obtain lower packet loss rate and provide more reliable transmission of realtime traffic information flow. Also as shown in Figs. 4 and 5, with the same VS number and moving speed, PLR of VoIP is always lower than that of Video, because the sent rate of VoIP is lower than that of Video, moreover, these above information flows are with the same traffic safety warning level, value of their traffic safety importance weights $\omega_{l}$ are of the same and has no effect on HOL packets’ scheduling order in all the buffers. The most important thing is shown in Fig. 6, data burst still obtain good throughput which more or less the same as that of the PF algorithm, for the reason that it adopts efficient adaptive modulation and coding technology and can obtain multi-user diversity gain. Thus, with the limited wireless resource, the proposed algorithm can still guarantee a good quality of data transmission for non-real-time information flow as well as real-time information flow. Certainly, as shown in Fig. 7, since PF algorithm factor $\psi_{n,k}^{\text{PF}} \left( t \right)$ is introduced into the proposed algorithm, it leads to the proposed algorithm only sacrifice little user fairness if compared with PF algorithm, which is a really low-cost but meaningful trade-off for effective resource allocation. As expected, both these two scheduling algorithms’ FI are very close to 1, and the proposed algorithm’s FI is slightly lower than that of the PF algorithm.

2. Scenario II: information flows with different traffic safety importance

Each VS terminal receives 3 information flows with the target transmission delay requirement $T_{s}$ = 100 ms. For *l* = 1, “highway.yuv” video test sequence is used to simulate video flow which closely related to traffic safety such as traffic accident report at the scene; For *l* = 2, “G.729” audio test sequence and FTP are respectively used to simulate VoIP flow and FTP which associating with travel entertainment, such as voice chat, document transmission, etc. Herein values of $\omega_{l}$ are respectively set to 0.8 for *l* = 1 and 0.4 for *l* = 2.

**Fig. 8:**
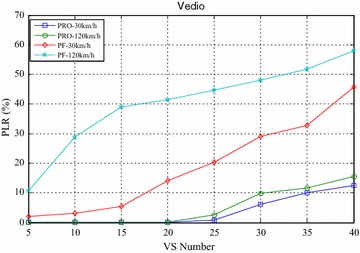
Packet loss rate of video

**Fig. 9:**
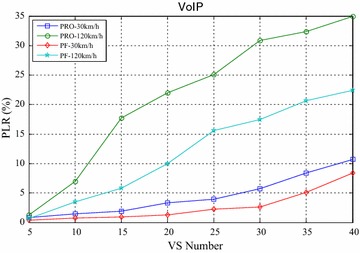
Packet loss rate of VoIP

**Fig. 10:**
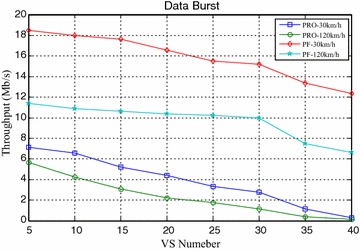
Throughput of data burst

**Fig. 11:**
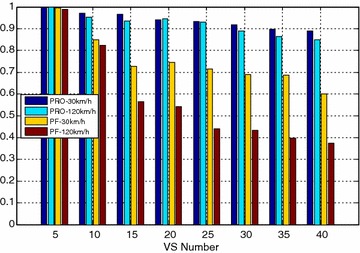
Fairness index of VS terminals

Figures 8 and 9 compared the packet loss rate (PLR) of video flow and VoIP flow respectively. As VS terminals’ number increasing, system traffic load is getting heavier and heavier, and packet loss rate is also increasing. When VS moving speed increasing, both video and VoIP flow’s PLR are growing for the same reason in the first simulation scenario. Since, the proposed algorithm has the ability to provide differentiated transmission services to those flows. It can adaptively adjust HOL packets’ scheduling order not only according to every HOL packet’s waiting time but also the information flows’ service type. Scheduler always selects the HOL packet which is highly related to traffic safety and also will be in violation of the delay limitation immediately, give it scheduling priority and deliver it as fast as possible. Obviously, the proposed algorithm is remarkable superior to the PF algorithm. Also as shown in Figs. 8 and 9, with the same VS number and moving speed, PLR of video is always lower than that of VoIP which is very different from their performance in the first scenario, because the key parameters $\omega_{l}$ are crucial for adjusting value of scheduling importance weight, and $\omega_{l = 1} > \omega_{l = 2}$ can make scheduler allocate more wireless resources to bear information flow which highly traffic safety. For the same reason, the proposed algorithm will sacrifice time non-sensitive information flow’s part of the ability for obtaining wireless resources, thus the data burst flow’s throughput is no more than that of PF algorithm as shown in Fig. 10. At the same time, VS terminals’ fairness index is further down in Fig. 11 if compared with that of the PF algorithm, because partial wireless resources are preferentially allocated to video and VoIP flows for meeting their comprehensive QoS requirements. However, these costs are the effective trade-off when the system needs to provide differentiated transmission services to different information flows.

## Conclusion

This paper proposes a novel downlink scheduling strategy for traffic communication system based on TD-LTE technology, which can perform two classification mappings for various information flows in the eNodeB: firstly, associate every information flow packet with traffic safety importance weight according to its relevance to the traffic safety; secondly, associate every traffic information flow with service type importance weight according to its QoS requirements. Once the connection is established, at every scheduling moment, scheduler would decide the scheduling order of all buffers’ HOL packets periodically according to the instant value of scheduling importance weight function, which calculated by the proposed algorithm. From different scenario simulations, it can be verified that the proposed algorithm can provide superior differentiated transmission service and reliable QoS guarantee to information flows with different traffic safety levels and service types, which is more suitable for traffic transportation environment compared with the existing popularity PF algorithm. With the limited wireless resource, information flow closed related to traffic safety will always obtain priority scheduling right timely, which can help the passengers’ journey more safe. Moreover, the proposed algorithm cannot only obtain good flow throughput and user fairness which are almost equal to those of the PF algorithm without significant differences, but also provide better realtime transmission guarantee to realtime information flow. However, it worth noting that if system number of VS terminals are too large and keep increasing, the limited wireless resources become hardly to bear system load, which will lead to performance continuously deteriorate no matter what schedule algorithm is adopted. Therefore, in our future work, we will consider how to set the proper user number and which communication technology can be a supplementary method to overcome that problem.
